# The Association of Iliac and Sacral Insufficiency Fractures and Implications for Treatment: The Role of Bone Scans in Three Different Cases

**DOI:** 10.7759/cureus.3861

**Published:** 2019-01-10

**Authors:** Sandeep Kola, Michelle Granville, Robert E Jacobson

**Affiliations:** 1 Physical Medicine and Rehabilitation, Larkin Community Hospital, Miami, USA; 2 Neurological Surgery, University of Miami Hospital, Miami, USA

**Keywords:** insufficency fractures, ilium, sacral fractures, sacroplasty, acetabulum rim fractures, osteoporosis

## Abstract

Iliac wing fractures are under-diagnosed fractures often associated with sacral insufficiency fractures in osteoporotic patients. They are rarely seen alone. Insufficiency fractures of the iliac bone can often be missed on computerized tomography (CT) and magnetic resonance imaging (MRI) yet identified on radioisotope bone scans. Symptomatic iliac fractures present with more lateralized pain in the hip and groin compared to patients with only sacral insufficiency fractures. Since the acetabulum is the key weight-bearing articulation between the sacrum and pelvis and the femoral head and leg, worsening of iliac stress fractures can have major effects on weight bearing and should be a consideration in patients with persistent pain in this area. The anatomy of the ilium and relationship to other pelvic insufficiency fractures is reviewed as well as treatment options. Typical cases are presented where the iliac fractures were found on bone scan either in addition to the more common sacral fracture or due to the persistence of symptoms of hip and thigh pain.

## Introduction

The iliac bone composes part of the pelvic ring and can be affected by both traumatic and osteoporotic sacral and pelvic fractures [1-2]. The iliac bone extends laterally, adjacent to the sacrum and the sacroiliac joint, to form the iliac crest on top of the iliac "blade" and then inferiorly to form the roof of the acetabulum [3-4]. Iliac fractures are rarely seen in isolation and almost 90% of patients found with iliac fractures have other lumbar and sacral fractures [1-3]. Traumatic fractures involving multiple pelvic bones are common with major trauma disrupting the pelvic rim leading to both anterior and posterior fractures often associated with diaphysis or spreading of the symphysis pubis [5-6]. Ilium fractures are also seen with relatively minor trauma in osteoporotic patients and patients who have received pelvic radiation [7]. In osteoporotic patients, attention has been generally focused on the sacrum and the different patterns of sacral insufficiency fractures seen with bone scans, and in the non-traumatic setting, imaging of the pelvic rim is not routinely performed and so bone scans may be the initial indication of abnormality in the ilium and acetabulum [8-9]. Osteoporotic fractures around the pelvic rim can be clinically insidious and are often found to be asymptomatic but also associated with more hip, groin and upper thigh pain [9]. Detection of these fractures is often based on radiologic findings such as identifying fractures with plain X-rays, isotope uptake in the ilium or acetabulum on radio-nucleotide bone scan, sclerosis and hypo-density with computerized tomography (CT), and edema with magnetic resonance imaging (MRI) [3,7-8]. MRI and CT abnormalities in the medial area of the ilium adjacent to the sacrum can be confused with metastatic diseases and so clinical context is important [10].

## Case presentation

Osteoporotic iliac fractures present in three distinct ways: 1) an asymptomatic finding associated with lumbar vertebral or sacral fractures, 2) a patient presenting with groin and hip pain, and 3) patients with severe hip pain and weight-bearing problems secondary to fracture involving the acetabular rim. An example case of each is presented.

Case 1

**Figure 1:**
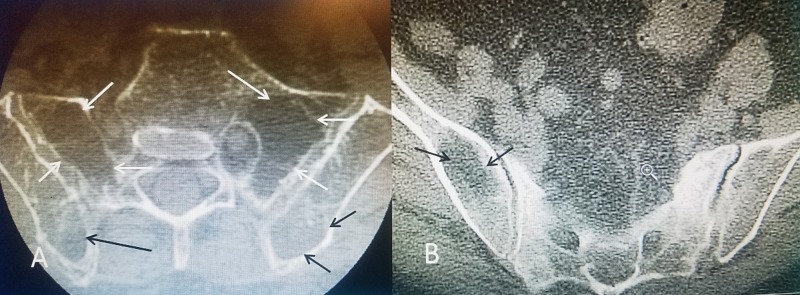
Axial CT showing sacral and iliac osteoporotic insufficiency defects A: CT scan of the sacrum and iliac wings showing severe osteopenia with loss of trabecula in the sacrum (solid white arrows) and similar but less severe change in the posterior iliac crest (solid black arrows); B: Loss of bone density in the middle part of the iliac wing more inferiorly (solid black arrows) CT: computed tomography

Detection of asymptomatic marked osteoporotic defects in the ilium on CT. The patient is a 73-year-old (y.o.) female with an eight-year history of multiple thoracic T8, T10, and L2 fractures and previous kyphoplasty, who presented with left paraspinal pain after a fall at home. She had been on alendronate for three years and her bone mineral density (BMD) 15 months prior was -2.4. Bone scan showed uptake at L4 and in the left sacral alae. CT scan of the lumbar spine, sacrum, and the pelvic rim showed marked osteopenia with the loss of trabecular pattern not only in the sacral alae but also in the iliac wings (Figure 1).

Case 2

**Figure 2:**
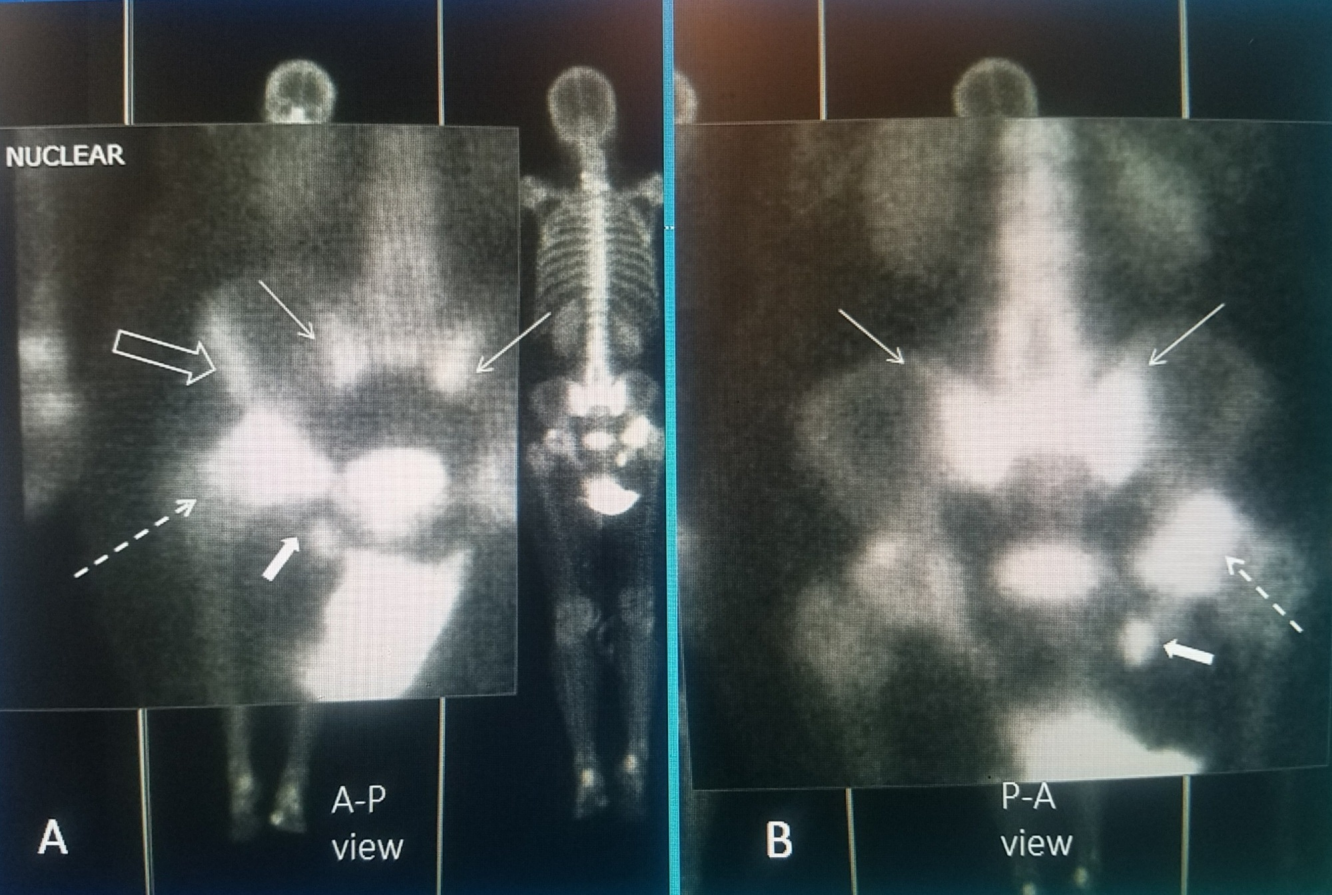
Magnified bone scan views showing a combination of bilateral sacral insufficiency fracture with complex multiple right ilium fractures A: Anterior-posterior (AP) view showing bilateral sacral insufficiency fractures with "Honda" sign (thin solid white arrow). There is a right ilium wing fracture (hollow white arrow), a large right acetabular rim fracture (dashed white arrow) and a small right ischium fracture (small white solid filled arrow); B: Posterior-anterior (PA) view showing the Honda sign is more prominent (thin white arrow) and the posterior acetabular rim fracture is seen (dashed white arrow) and small ischium fracture (filled solid white arrow). The ilium wing fracture is not seen since it is more anterior.

Bilateral sacral insufficiency fractures with unilateral acetabular rim fracture and smaller ischium fracture. The patient is a 78 y.o. female with a long history of chronic corticosteroid use for Sjogren's syndrome for over 12 years. She has had multiple thoracic and lumbar fractures treated at different times with four kyphoplasty and vertebroplasty procedures over eight years. She was on alendronate and later teriparatide daily injection for 24 months. Initially, she responded well to the procedures with the loss of pain and return to full activity but gradually developed progressive lumbar-thoracic kyphoscoliosis, leading to chronic back pain. She was treated with the addition of a thoracolumbosacral orthosis (TLSO brace). She then started complaining of low lumbosacral pain, bilaterally as well as left thigh and later severe left hip pain that worsens on weight-bearing. Five years previously, she had a right knee replacement. There were no new falls and the initial X-rays of the hip showed mild degenerative osteoarthritis. Bone scan showed bilateral sacral alae uptake and marked uptake in the rim of the acetabulum in the area of her hip pain that was initially felt to be a possible femoral head osteonecrosis, but MRI of the hip showed minimal subchondral bone erosion and acetabular narrowing and the bone scan was interpreted as consistent with the right acetabular rim and ischium fracture (Figure 2).

Case 3

**Figure 3:**
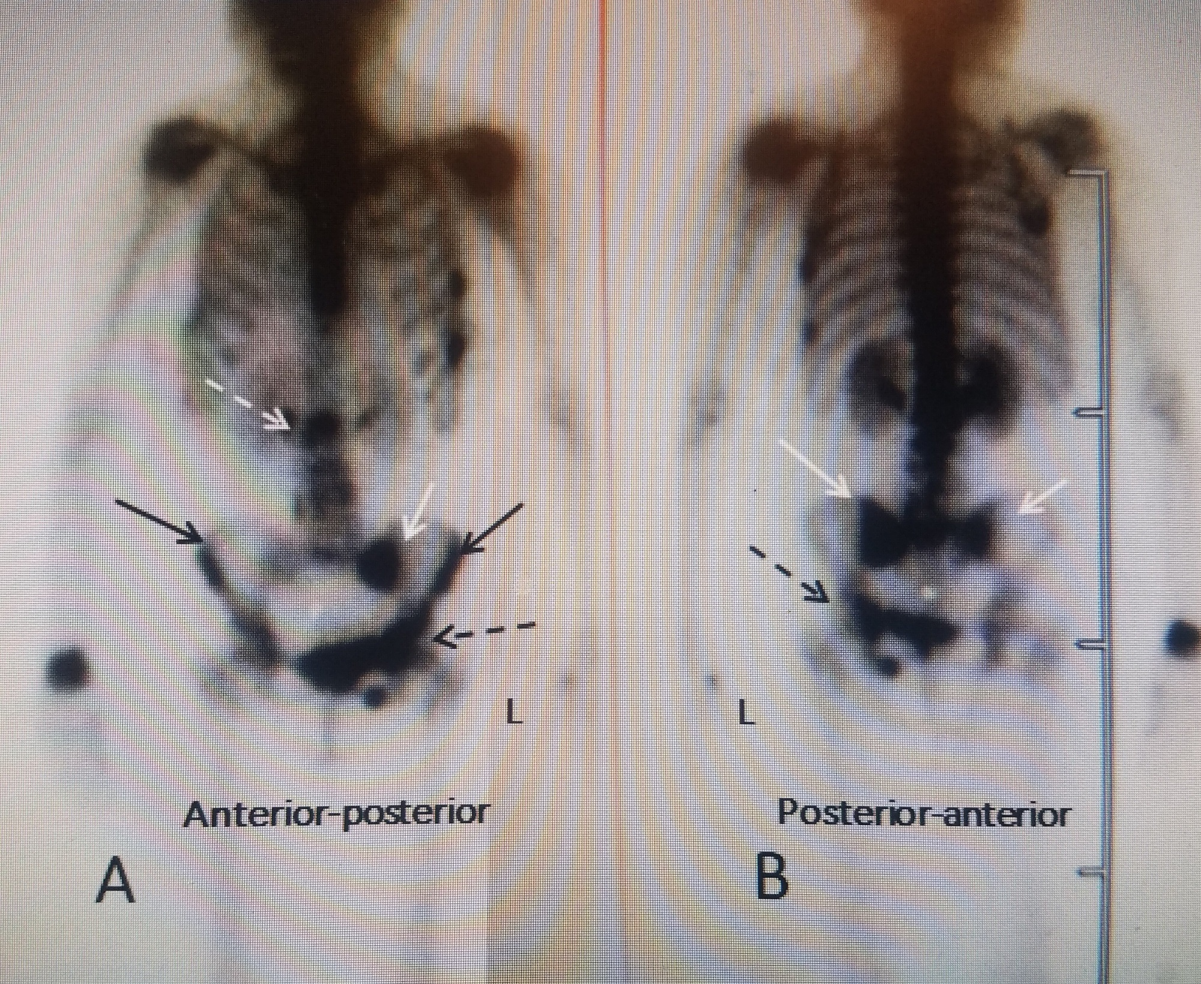
Bone scan demonstrating multiple sacral and left iliac fractures A: Anterior-posterior (AP) views demonstrating multiple pelvic rim fractures in an 81-year-old female with osteoporosis: a T12 fracture (dashed white arrow), left (L) sacral alae fracture (solid white arrow), bilateral iliac wing fractures (solid black arrow), and left ischium, symphysis pubis and acetabular fractures on the left (dashed black arrow); B: Posterior-anterior view showing that the sacral fracture is bilateral (solid white arrow) and again the left ischium and acetabular fracture are identified (dashed black arrow).

Osteoporotic fractures in the upper lumbar spine, bilateral sacral fractures and multiple fractures in the iliac wings, acetabular, and the ischium. The patient is an 81 y.o. female who was complaining of low back and lumbosacral pain to the left lateral groin and upper thigh, which worsened on weight bearing. She had never been on any treatment for osteoporosis and initial BMD was -2.7. She had been at prolonged bed rest as a result of previous left knee arthroplasty and thrombophlebitis. While undergoing inpatient rehabilitation and beginning weight-bearing ambulation within three days, she developed severe groin pain. Plain X-rays only showed degenerative spondylosis of the lumbar spine, mild spondylolisthesis at L4-5 and mild narrowing of the acetabular joints bilaterally. A bone scan showed positive isotope uptake at T12, bilateral sacral uptake with an incomplete "Honda" sign worse on the left and multiple areas of marked uptake along the left iliac wing continuing into the acetabular rim and ischium (Figure 3).

## Discussion

**Figure 4:**
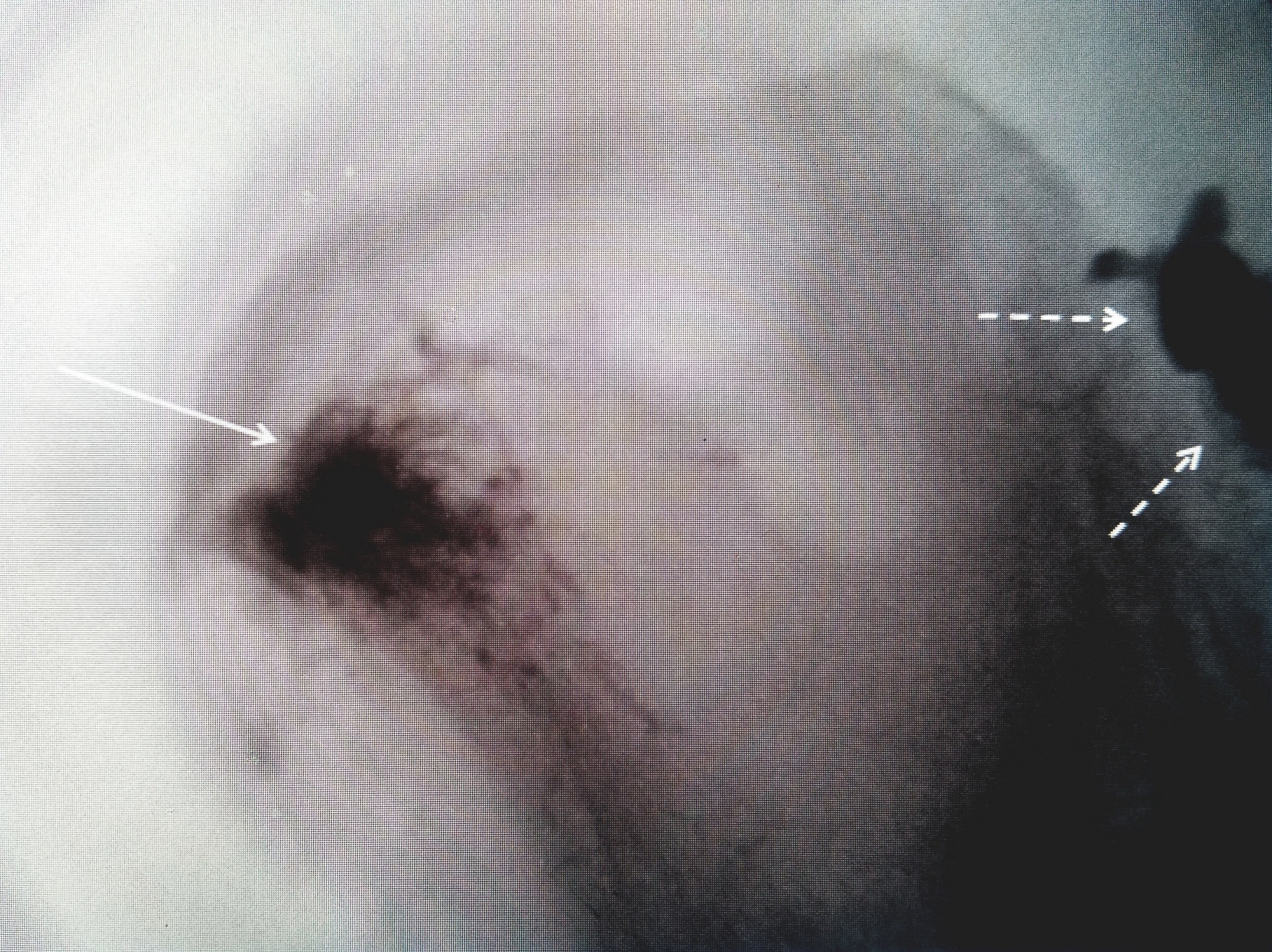
Use of bone cement for symptomatic iliac crest fracture Iliac insufficiency fracture (solid white arrow) injected for persistent pain after sacroplasty (dashed white arrows)

Iliac insufficiency fractures can be an associated finding in the evaluation of patients with sacral insufficiency fractures. It indicates more extensive osteoporosis and disruption of the pelvic ring with potential biomechanical consequences. A distinction needs to be made between the "pelvis" and the "pelvic girdle", as the two are often used interchangeably. The pelvic girdle, or Os Coxae, consists of the fused bones identified as the ilium, ischium, and the pubis. The "girdle" from each side is joined together anteriorly by the pubic symphysis and posteriorly at the sacrum [1,3-4]. Biomechanically, the pelvis functions both as support for the lumbar spine and a center for both hip or femoral heads to articulate at the acetabulum which is the lower part of the ilium. It is known that sacral insufficiency stress fractures occur at the intersection of load bearing between the axial spine and the sacral alae [11]. This same mechanical load and stress can extend to the thinned ilium bone and acetabular rim and is also more common when there is an abnormality of motion of the hip or knee commonly seen with a joint replacement that further alters the load on the sacral-iliac region [11]. Traumatic fractures of the anterior pelvic rim, especially involving the roof of the acetabulum, may require internal fixation or unilateral arthroplastic joint replacement [12-14]. Sacral osteoporotic insufficiency fractures can be treated with bone cement or sacroplasty and similarly injection of bone cement into the associated ilium fracture may be required for persistent pain in a fracture area after conservative treatment (Figure 4).

The detection of pelvic girdle fractures can be difficult. Radiologic studies often do not include the pelvic ring unless specifically requested, and so unless diaphysis of the symphysis pubis or an ischium fracture is seen on plain X-rays, there may not be an obvious indication of ilium fractures. Bone scans can not only detect lower vertebral fractures and sacral insufficiency fractures but as shown in these case examples unsuspected single or multiple ilium fractures [15-18]. These fractures should be considered whenever there is more lateralized hip or thigh pain or atypical pain. In the early stages, a bone scan may be a more sensitive radiologic examination, especially in patients with unclear clinical symptoms. Since the radiologic focus is initially directed at the lower lumbar spine and sacrum attention must be directed to the adjacent ilium, ischium, and the acetabular ring if the bone scan reveals associated fractures in these areas [17-20]. These fractures probably occur more frequently than reported since unless a bone scan is performed they may not be detected. The finding associated ilium fractures with lower lumbar and sacral insufficiency fractures indicates that the pelvic rim is more severely affected than originally suspected and may explain persistent clinical symptoms in some patients after vertebroplasty or sacroplasty.

## Conclusions

Many iliac fractures are detected on bone scan or MRI without symptoms but almost always in association with other pelvic sacral fractures. Patients presenting with more lateralized hip and thigh pain with a lumbar or sacral insufficiency fracture should be evaluated for the presence of one or more ilium fractures. The typical patient is more elderly, with a long history of intermittently treated osteoporosis, yet with a high negative BMD score, a history of previously treated osteoporotic spinal fractures, commonly previous knee or hip surgery or arthroplasty, all leading to increased mechanical stress on the weakened sacrum and ilium bones. Generally, ilium fractures do not require specific treatment, or at most additional use of bone cement into the ilium when near the sacrum. Acetabular fractures can be different and can have a profound effect on weight bearing and so they may need either open surgical fixation with or without cement stabilization or ultimately if progressive, hip joint arthroplasty.

## References

[1] Davies AM, Bradley SA (1991). Iliac insufficiency fractures. Br J Radiol.

[2] Linstrom MJ, Heiserman JE, Kortman KE (2009). Anatomical and biomechanical analyses of the unique and consistent locations of sacral insufficiency fractures. Spine.

[3] Rizzo PF, Gould ES, Lyden JP, Asnis SE (1993). Diagnosis of occult fractures about the hip. Magnetic resonance imaging compared with bone-scanning. J Bone Joint Surg Am.

[4] Hernigou J, Alves A, Homma Y, Guissou I, Hernigou P (2014). Anatomy of the ilium for bone marrow aspiration: map of sectors and implication for safe trocar placement. Int Orthop.

[5] Alton TB, Gee A (2014). Classifications in brief: Letournel classification for acetabular fractures. Clin Orthop Relat Res.

[6] Na WC, Lee SH, Jang HW, Jo S (2017). Pelvic insufficiency fracture in severe osteoporosis patient. Hip Pelvis.

[7] Oh D, Huh SJ (2014). Insufficiency fracture after radiation therapy. Radiat Oncol J.

[8] Cabarrus M, Ambekar A, Lu Y, Link T (2008). MRI and CT of insufficiency fractures of the pelvis and the proximal femur. AJR Am J Roentgenol.

[9] Kanberoglu K, Kantarci F, Cebi D (2005). Magnetic resonance imaging in osteomalacic insufficiency fractures of the pelvis. Clin Radiol.

[10] Donovan A, Schweitzer ME, Rafii M, Lax A (2009). Radiologic features of superomedial iliac insufficiency fractures: a possible mimicker of metastatic disease. Skeletal Radiol.

[11] Linstrom NJ, Heisermann JE, Kortman KE (2009). Anatomical and biomechanical analysis of the unique and consistent locations of sacral insufficiency fractures. Spine.

[12] Vanderschot P (2007). Treatment options of pelvic and acetabular fractures in patients with osteoporotic bone. Injury.

[13] Andreas Höch A, Pieroh P, Henkelmann R, Josten C, Böhme J (2017). In-screw polymethylmethacrylate-augmented sacroiliac screw for the treatment of fragility fractures of the pelvis: a prospective, observational study with 1-year follow-up. BMC Surg.

[14] Pagebnkopf E, Grose A, Partal G, Helfet D (2006). Acetabular fractures in the elderly: treatment recommendations. HSS J.

[15] Kim YY, Chung BM, Kim WT (2018). Lumbar spine MRI versus non-lumbar imaging modalities in the diagnosis of sacral insufficiency fracture: a retrospective observational study. BMC Musculoskelet Disord.

[16] Bakker G, Hattingen J, Steutzer H, Isenberg J (2018). Sacral insufficiency fractures: how to classify?. J Korean Neurosurg Soc.

[17] O’Connor TJ, Cole P (2014). Pelvic insufficiency fractures. Geriatr Orthop Surg Rehabil.

[18] Lawrence D, Kirsten Menn K, Baumgaertner M, Haims A (2013). Acetabular fractures: anatomic and clinical considerations. AJR: Focus on Musculoskeletal imaging.

[19] Oberkircher L, Ruchholtz S, Rommens PM, Hofmann A, Bücking B, Krüger A (2018). Osteoporotic pelvic fractures. Dtsch Arztebl Int.

[20] Henry PD, Kreder HJ, Jenkinson RJ (2013). The osteoporotic acetabular fracture. Review article. Orthop Clin North Am.

